# *POLQ* in Breast Cancer

**DOI:** 10.18632/oncotarget.120

**Published:** 2010-07-11

**Authors:** Adrian Begg

**Affiliations:** Division of Experimental Therapy, The Netherlands Cancer Institute, Amsterdam

Expression profiling has produced gene signatures prognostic for outcome in a number of cancers, with breast cancer heading the list in terms of numbers of studies and progress towards clinical use. Some of these signatures, which contain multiple genes, have been validated in several independent clinical series and have been approved for the clinic. Their usefulness lies not only in their ability to predict if a patient will die early or late or hopefully not at all, but also to spare patients from toxic treatment, e.g. chemotherapy, if it will not benefit them. The search for prognostic factors therefore represents more than just an academic exercise. Equally valuable would be to predict which treatment would the most effective against the tumor.

In this issue of *Oncotarget*, Higgins and colleagues [1] describe another prognostic gene signature for breast cancer, this one being particularly unusual in containing just a single gene, *POLQ* (official name: polymerase DNA directed theta). Their stimulus to study this gene came from a recently published paper from their lab in which *POLQ* emerged from siRNA screening for genes determining radiosensitivity [2]. The exact role of this gene is still not entirely clear, although it has been reported to function in pathways dealing with DNA lesions, including base excision repair and translesional synthesis, consistent with a role in sensitivity to ionizing radiation.

Given this finding, they naturally wanted to know if it had any clinical relevance, hence their study of several clinical series in which they or others had measured global gene expression using microarray technology. Breast cancers were an obvious choice given the amount of publically available expression data. Gratifyingly, *POLQ* turned out to be a strong prognostic marker in all but one of the series they studied, and even in this series it showed a strong trend. Several other gene signatures have been discovered and validated to have prognostic significance for breast cancer, so is *POLQ* better or different? They report that *POLQ* appears in some of the published signatures but not in others. By looking at genes co-regulated with *POLQ*, they also showed that many of the molecular pathways represented by these genes also occurred in the published signatures. Given the large difference in outcome found between high and low *POLQ* expressing tumors, the authors speculate that *POLQ* may be one of the most important driver genes in other signatures which contain *POLQ*. Further, they found that cyclin E (*CCNE2*), amazingly, was the only common gene between all three of the main published prognostic breast cancer signatures and the set of genes positively correlating with *POLQ* expression. Pursuing this, they could show that *CCNE2* was also prognostic by itself, independently of *POLQ*, and that the two genes together (*POLQ* and *CCNE2*) had even stronger prognostic power.

While this is highly interesting and potentially useful, it would be even more useful to know why *POLQ* is prognostic, since only then can rationally chosen targeted interventions be employed for patients with high *POLQ* expressing tumors. So does *POLQ* simply monitor radiosensitivity, which would then suggest the use of a radiosensitizer strategy? This is probably unlikely since some patients in their series (around 17%) did not receive radiotherapy, while all received surgery, and the majority received chemotherapy and hormone therapy. Given the magnitude of the *POLQ* effect, it is more likely, as speculated by the authors, that this gene provides a measure of general malignancy, related to high proliferation status, defective p53 signaling, high tumor grade and negative ER status. Endpoints used in the clinical studies also included metastases, and so it is possible that *POLQ* could also be correlated with invasive and other properties of tumor cells rendering them more capable of metastasizing. This reasoning also applies to *CCNE2*. All this needs further study, including a breakdown of the data into different endpoints and the different treatments.

On a practical note, the finding that one or two genes have such strong prognostic value suggests the use of robust and routine assays such as immunochemistry or PCR-based methods which can be carried out rapidly in the majority of centers. Such methods should also facilitate further validation on independent patient series. Finally, given these interesting findings, one of the next questions is whether *POLQ* and/or *CCNE2* also have prognostic significance in other cancers. I expect we will hear about this in the near future and we await such results with interest.
